# Consequences of Laughter Upon Trunk Compression and Cortical Activation: Linear and Polynomial Relations

**DOI:** 10.5964/ejop.v12i3.1102

**Published:** 2016-08-19

**Authors:** Sven Svebak

**Affiliations:** a Faculty of Medicine, NTNU, Norwegian University of Science and Technology, Trondheim, Norway; Department of Psychology, University of Western Ontario, London, Canada

**Keywords:** cortical activation, laughter, linear, polynomial, trunk compression

## Abstract

Results from two studies of biological consequences of laughter are reported. A proposed inhibitory brain mechanism was tested in Study 1. It aims to protect against trunk compression that can cause health hazards during vigorous laughter. Compression may be maximal during moderate durations and, for protective reasons, moderate in enduring vigorous laughs. Twenty-five university students volunteered to see a candid camera film. Laughter responses (LR) and the superimposed ha-responses were operationally assessed by mercury-filled strain gauges strapped around the trunk. On average, the thorax compression amplitudes exceeded those of the abdomen, and greater amplitudes were seen in the males than in the females after correction for resting trunk circumference. Regression analyses supported polynomial relations because medium LR durations were associated with particularly high thorax amplitudes. In Study 2, power changes were computed in the beta and alpha EEG frequency bands of the parietal cortex from before to after exposure to the comedy “Dinner for one” in 56 university students. Highly significant linear relations were calculated between the number of laughs and post-exposure cortical activation (increase of beta, decrease of alpha) due to high activation after frequent laughter. The results from Study 1 supported the hypothesis of a protective brain mechanism that is activated during long LRs to reduce the risk of harm to vital organs in the trunk cavity. The results in Study 2 supported a linear cortical activation and, thus, provided evidence for a biological correlate to the subjective experience of mental refreshment after laughter.

Many resent observations and studies have reported beneficial as well as harmful effects of laughter upon health. A recent review of 785 papers published on laughter over more than 65 years (Ferner & Aronson, 2013) reported benefits (85 reports) as well as harms from laughter (114 reports). They included psychological benefits, such as reduced anger, anxiety, depression and stress, but also cardiovascular (improvement of endothelial function), respiratory (improved lung function), metabolic (increase of energy expenditure) and obstetric effects: Entertainment by a clown after embryo transfer increased successful fertilization with 16% (Friedler et al., 2011). Harmful effects of laughter included cardiovascular (syncope, arrhythmias, cardiac rupture) and respiratory effects (asthmatic attack, pneumothorax, interlobular emphysema), as well as effects upon the central nervous (cataplexy, cerebral infarct), gastrointestinal (hernia), musculoskeletal (dislocation of jaw) and urinary (stress incontinence) systems.

Despite the common idea that laughter is good for your health, trunk compression during vigorous laughter may escalate to levels of high internal pressure that can cause health hazards. This more complex idea was indicated as a buffer against excessive pressure in the thorax cavity needed during intense laughter to protect vital organs from harm (Filippelli et al., 2001). Support to this protective mechanism was expected in Study 1 by maximal compression during laughter responses (LR) of medium duration and that compression is moderate in short as well as long durations. In this way, vital organs would be protected from harms induced by forceful trunk compression during vigorous and enduring LRs. A linear relation between vigorous laughter and trunk compression would involve the increasing risk of harm with increasing duration of laughs.

Hilarious laughter typically is followed by a subjective state of refreshment. The second study to be reported here, assessed consequences of laughter upon cortical activation as indicated by parietal EEG changes from pre to post exposure to comedy. A linear relation between indices of cortical activation and duration as well as frequency of laughter was expected. In this way, Study 2 tested a possible cortical correlate of the subjective experience of refreshment *after* laughter, whereas the focus in Study 1 was upon biological consequences *during* laughter.

One challenge in research on laughter is the reliability of the method used for the assessment of a LR. The most obvious approach would be to count vocal sounds that are intuitively recognized by the observer as laughter. However, this approach is not sensitive to laughter that sometimes, for social or personal reasons, may occur with marked trunk compression without being accompanied by any vocal expression or by any obvious facial sign of amusement. Furthermore, participant observation is likely to be inaccurate in defining the onset, intensity and termination of a laughter response. However, this approach may still be useful in studies of laughter in everyday life and was recently termed “sidewalk neuroscience” (Provine, 2016). Participant observation has also been an adequate basis for assessment in patients with syncope following laughter (see e. g. Kim & Frishman, 2012) and in pathological laughter due to brain damage (Lauterbach, Cummings, & Kuppuswamy, 2013).

Neural and motor correlates of smiling and laughter have been studied by the use of a range of laboratory techniques in recent years, including the assessment of diaphragm movements by electromyography (EMG; e. g. Kimata et al., 2009), sometimes in combination with chest wall surface EMG (see review by Luo, Moxham, & Polkey, 2008), sometimes applied in combination with cervical magnetic and electrical phrenic nerve stimulation (e. g. Luo et al., 1998). Most of these techniques are invasive and ideal for use in clinical settings, but have questionable reliability when applied in settings when mirthful laughter is to be studied in the laboratory. This view extends to the use of functional magnetic resonance imaging in the investigation of brain neural correlates of laughter (e. g. Wattendorf et al., 2013).

In the present studies, laughter was scored by mercury-filled strain gauges that have been proven to be highly sensitive to trunk circumference changes and hardly noticeable by the subject when in use. Svebak (1975, 1982, 1985) presented criteria for the operational scoring of LRs, and they were applied to identify instances of laughter in both studies.

## Study 1

Respiratory alterations are involved in the expression of all intense emotions. Thus, Bloch, Lemeignan, and Aguilera (1991) reported that specific patterns distinguish among the intense human expressions of joy-laughter, sadness, fear-anxiety, anger, erotic love and tenderness. In their research, respiration movements were measured by means of a strain gauge transducer attached to a slightly elastic belt around the waist. Therefore, their research could not address the relative importance of the thorax and the abdomen nor the overall compression in the expiration phase of emotional expression. However, they concluded that for anger, erotic love and tenderness, significant changes in amplitude, rate and duration of the expiratory pause were crucial, whereas the differentiation of joy, sadness and fear was best achieved by the inspiration over expiration time ratios and were further characterized by small amplitudes as well as high rate saccadic movements. The risk of harmful pressure in the trunk cavity during expression was not at focus in their research.

The technologically advanced study by Filippelli et al. (2001) applied a set of optoelectronic plethysmography sensors distributed across the front and the back, at seven levels of the trunk, in eleven subjects to three-dimensionally trace their trunk movements during laughter with infrared cameras positioned at the front and the back. They also applied pressure sensors and respiratory flow measures. They concluded that all fits of laughter were characterized by a sudden occurrence of expiratory movement, and that the upper and lower sections of the thorax, as well as the abdomen, contributed to the decrease in lung volume. Their findings supported a significant role for the thorax muscles in the sudden increase of pressure upon all organs in the upper trunk, including the pleura cavity, during laughter. They reasoned that the diaphragm acts as a protecting buffer to the thorax cavity during laughter. However, their technology did not permit a relative comparison of the importance of the thorax and the abdomen in the production of moderate to intense expressions of laughter, taken that they did not measure trunk circumference changes with individually calibrated percentages of compression movements. And their data were analyzed by linear, and not by polynomial tests.

Specifically, the possible inhibition of trunk compression in the thorax during long and hilarious laughter may act to protect the heart, blood vessels and lungs from harms, whereas inhibition of the belly compression may act to reduce the risk of incontinence and hernia. In Study 1, the frequency and duration of LRs were calculated as well as the trunk compression (amplitude) in every LR, relative to the resting circumference, in the abdominal and thoracic sections of the trunk.

The limited research on trunk involvement in laughter implies that predictions in the present research could not be based upon previous findings. In Study 1, the trunk movements, rather than the subjective experience of amusement, was at focus to test the hypothesis of a protective mechanism that may involve the thorax as well as the abdomen. Possible gender differences in LR compression were tested for exploratory reasons.

It was assumed that evidence in support of a linear relation between trunk compression and the duration of LRs would fail to support the idea of a protective brain mechanism that is activated during the longest LRs. In contrast, a polynomial (quadratic) relation between LR-induced trunk compressions and the duration of LRs would present support to the activation of a protective brain mechanism during particularly long LRs. Compression in the thorax and the abdomen were tested separately in the present study.**^i^**

### Method

#### Participants

Twenty-five students volunteered. They were recruited from lectures in introductory university courses in social sciences, one by one, to view an entertainment video program in the laboratory. Mean age was 21 years (range: 19-32), and they were balanced across gender (13 females). They all were recruited at the end of a lecture class and were not compensated for their participation.

#### Apparatus

**Figure 1:**
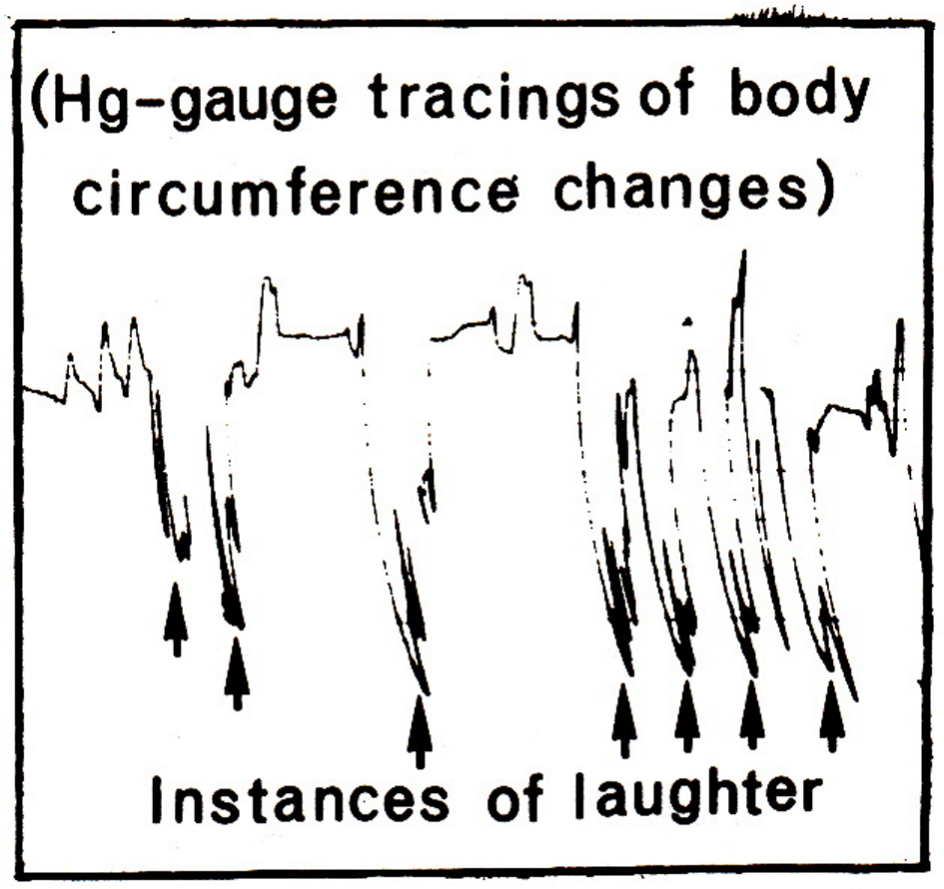
Illustration of trunk circumference changes (compressions) during laughter induced by a video entertainment program (“Dinner for one”). Changes are traced by a mercury-filled strain gauge strapped around the abdomen, and regular breathing amplitudes are indicated to the far left in the figure. Arrows indicate the occurrence of laughter.

The technology for measuring trunk circumference changes was identical to what has been described in previous reports (Grossman & Svebak, 1987; Svebak, 1975, 1982). A Beckman R Dynograph recorded trunk circumference changes, with two 9875B couplers where the time constant switch was set to the DC position. A case illustration of the tracings of the abdomen is given in Figure 1.

Mercury-filled Hg-gauges were made of silicone tubes with 0.5 mm inside diameter and 0.925 outside diameter. They were filled with standard triple distilled mercury, and platinum wires (1.125 mm diameter) of 2.5 cm were inserted about 8 mm into each end of the silicone tubes. The Hg-gauges were plugged into a T-shaped double pin holder that was connected to the preamplifier of the dynograph.

The length of a Hg-gauge was chosen according to the circumference of the thorax, at the level of the armpits, and the abdomen (half way between the lowest point of the rib bones and the highest point of the hip bone) when the subject was seated. In this way, all gauges operated at a stretch of between 25 and 35% added to its resting length. This procedure assured reliable recordings of resistance changes during laughter. Svebak (1986) documented the high sensitivity of these gauges for tracing trunk circumference changes.

#### Materials and Procedure

The entertainment material was composed of a twenty-five minute video presentation based on episodes from a series of candid camera presentations on Norwegian TV, recommended by the producer based upon which episodes turned out to be the funniest according to feedback from the viewers.

The participants were invited to the laboratory, one by one, a couple of days before recording, and informed by the experimenter about the laboratory setting, technical equipment and procedure. This orientation provided the establishment of an informal social relation between the participant and the experimenter. On the recording day, the participant was seated in front of the TV screen, two meters away, and mercury strain gauges were strapped around the thorax and the belly. The recording session started with a resting period of ten minutes where the participant was instructed to relax with closed eyes, and resting respiration was sampled over the last minute of this period. Then the experimenter entered and presented a brief description of the entertainment program.

The social setting was facilitated by the use of headphones that provided a sound track and dubbed laughter to the participant, and the experimenter was seated one meter to the left. He also was equipped with headphones when watching the entertainment program. However, no sound was provided in these headphones and, instead, they gave the impression to the participant that the experimenter listened to the TV program. There was no emitted sound directly from the TV, but only via the headphones to the subject. This arrangement permitted the experimenter to listen to vocalizations from the participant and, in each case, take care to laugh about as often and as intensely as the participant. Before start of the entertainment program, the participant was informed about a debriefing interview at the end of the program, where they could report their evaluation. The laboratory session ended with the unstructured debriefing interview.

#### Data Scoring and Statistics

Data were scored according to criteria given in previous publications (e. g., Svebak, 1975, 1982). Trunk circumference changes were calculated in percent as a fraction relative to the mean smallest abdominal and thoracic circumferences, respectively, over the initial one-minute resting period. A LR was scored as a sudden reduction in circumference, at least twice the mean amplitude of the resting respiration cycles, and the duration of a LR ended when the trunk circumference suddenly returned to the initial level (see Figure 1). This scoring procedure was dependent upon the establishment of a metric scale in both trunk segments and for every subject. Thus, when the subject had left, the two gauges that had been in use, were stretched stepwise, one by one cm, until pen deflection defined the whole scale for the operating pen deflection and for each channel (Svebak, 1986). HA-responses were calculated as the number of oscillations per second at the bottom of the LR-amplitudes (see Figure 1), and scores were given for each trunk level as the mean number per second for the five longest LRs.

Scores were analyzed by use of the SSPS-21 statistical package with t-tests (gender differences), product-moment correlations, as well as linear and polynomial regression analyses.

### Results

Descriptive statistics are given in Table 1. The mean number of LRs was 97.7 and their mean duration was 4.4 seconds. On average, the abdominal circumference was reduced by 1.4 percent during a LR, and the corresponding score for the thorax was 1.9. This difference in trunk amplitude was significant (paired samples test: *t*(24) = 3.51, *p* < .002). The frequency of HA-responses was 3.4 per second in the abdomen and 3.6 in the thorax (paired samples test: *t*(24) = 0.51, n. s.). A significant gender difference was seen for the abdominal LR compression amplitudes, and for the HA-response frequencies in the abdomen as well as thorax, with high scores in the males (see Table 1).

**Table 1:** Descriptive Statistics for Laughter Responses (LR) and Their Trunk Parameters^a^

Variables	Mean	Range (Std.)	Sex (M/F)	Sex diff. (*p*-value)	95% CI
Total LR	97.7	5 - 197 (52.3)	95.3/99.9	0.21 (n.s.)	[-42.38, 41.67]
Duration of LR	4.4	0.6 - 9.2 (2.3)	4.0/4.7	0.69 (n.s.)	[-2.87, 0.75]
Abd. LR amplitude^a^	1.4	0.2 - 3.0 (0.8)	1.8/1.0	2.81 (<.010)	[0.22, 1.43]
Thor. LR amplitude^a^	1.9	0.4 - 2.3 (0.8)	2.2/1.7	1.65 (n.s.)	[-0.13, 1.14]
Abd. HA-frequency	3.4	0.0 - 6.3 (1.7)	4.5/2.5	3.73 (<.001)	[0.83, 3.17]
Thor. HA-frequency	3.6	0.0 - 5.9 (1.6)	4.4/2.9	2.71 (<.013)	[0.36, 2.69]

*Note.* Trunk parameters = abdominal and thoracic compression, i. e. circumference reduction, during LR; abdominal and thoracic HA-responses per second. Means for males (M) and females (F) and related t-scores for sex differences are included with *p*-values (df = 23). Confidence intervals are included (95% CI).

^a^Calculated as a percentage of the mean resting circumference at the end of the expiration phase for the abdomen and the thorax, respectively.

**Figure 2:**
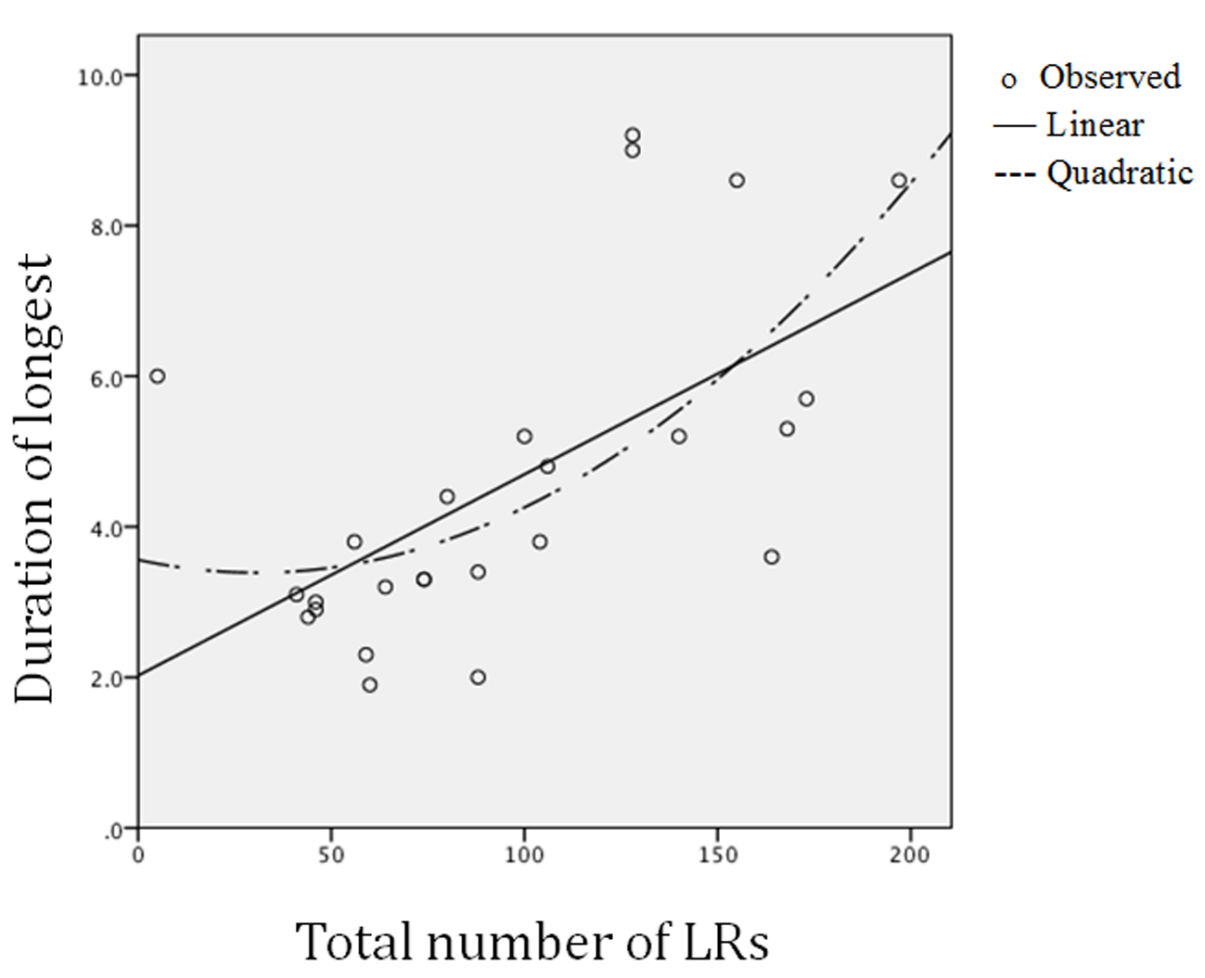
Linear and polynomial relations between (a) total number of laughter responses (LRs) and (b) mean duration of the five longest LRs.

The relation between total number and duration of LRs was tested by linear regression (see Figure 2) and yielded a significant standardized beta score of 0.60 (*p* < .001), whereas the polynomial test presented a non-significant beta (-0.25). Pair-wise linear trends between all variables except sex are given in Table 2 where total LRs were positively correlated with the LR amplitudes of the thorax. The mean duration of the five longest LRs was not significantly associated with other variables. The compression amplitudes of LRs in the abdomen and thorax were positively correlated, and they were also positively correlated with the abdominal HA-frequency scores. HA-frequencies were high in the thorax when they were high in the abdomen.

**Table 2:** Correlations Between Total Number of Laughter Responses (LR), Mean Duration of the Five Longest LRs, Trunk Variables in the Abdomen and Thorax (Compression, i. e. Circumference Reduction During LR) and Frequency of Trunk HA-Responses.

Variables	1	2	3	4	5	6
1. Total LR	–	.80***	.26	.49**	.20	.06
2. Duration of LR		–	.23	.26	.09	-.09
3. Abd. LR amplitude			–	.56**	.53*	.34
4. Thor. LR amplitude				–	.55**	.46*
5. Abd. HA frequency					–	.71***
6. Thor. HA frequency						–

*p < .05. **p < .01. ***p < .001 (*df* = 23; two-tailed tests).

**Figure 3:**
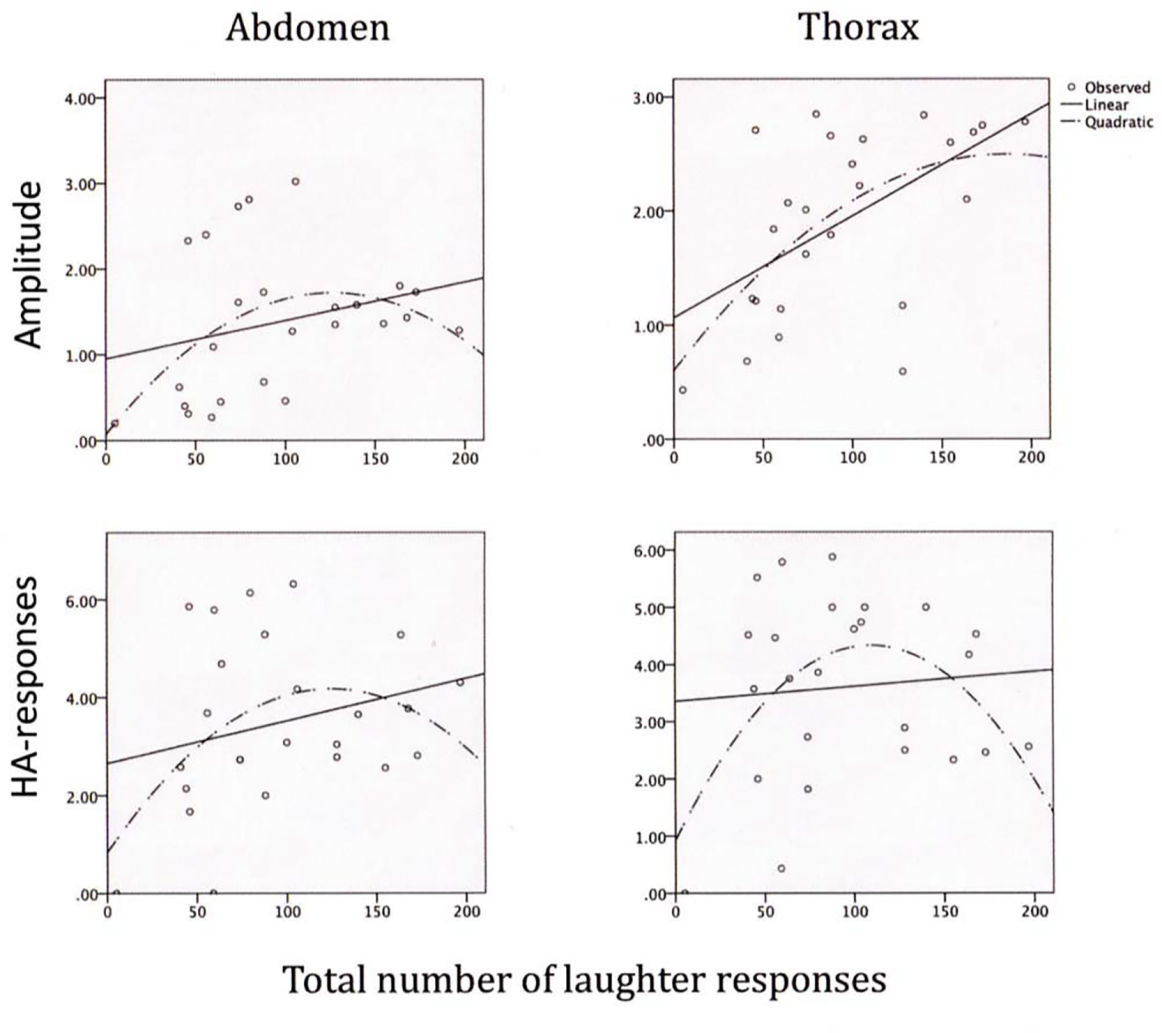
Linear and polynomial relations between (a) the total number of laughter responses and (b) abdominal and thoracic compression amplitudes during laughter (upper panels) as well as frequencies of abdominal and thoracic HA-responses (lower panels).

Tests on linear and polynomial trends for the LR scores with the trunk compression amplitude and HA-frequency scores are given in Table 3 (upper section) and illustrated in Figure 3. The scores for the linear trends of the LR frequencies with the trunk parameters were mostly non-significant, and the 95% confidence interval included the zero in all cases but one (thorax LR-amplitudes). In contrast, the polynomial betas for the number of LRs with the thorax amplitudes and HA-responses were significant, and they were nearly significant for the abdominal scores. This meant that the LR compression amplitudes were high (abdomen: around 1.5%; thorax: around 2%; see upper panels of Figure 3) with moderate numbers of LRs, and the HA-frequencies were high (around 4/sec. in the abdomen as well as the thorax) with moderate numbers of LRs (around 120 over the 25 minutes). Both low and high numbers of LRs were associated with lower compression amplitudes as well as HA-frequencies in the abdomen as well as the thorax.

**Table 3:** Linear and Polynomial Analyses (Standardized beta: B) of Relations Between Laughter Responses (LR; Upper Panel: Frequencies; Lower Panel: Duration) and the Related Abdominal and Thoracic Compression (Circumference) Parameters. Confidence Intervals Are Included (95% CI).

	Linear				Polynomial	
Parameters	*B* (1/23)	*p*	95% CI	*B* (2/22)	*p*
Total number of LRs by:					
Abd. LR amplitude	0.21	n.s.	[-9.66, 42.55]	1.65	.071
Thor. LR amplitude	0.49	.013	[7.59, 57.08]	1.84	.028
Abd. HA frequency	0.20	n.s.	[-12.29, 16.17]	1.74	.060
Thor. HA frequency	0.06	n.s.	[-6.74, 18.83]	2.22	.014
Mean duration of five longest LRs by:					
Abd. LR amplitude	0.23	n.s.	[-0.53, 1.81]	2.16	.017
Thor. LR amplitude	0.26	n.s.	[-0.46, 1.98]	3.23	.0004
Abd. HA frequency	0.09	n.s.	[-0.76, 0.50]	1.95	.035
Thor. HA frequency	0.09	n.s.	[-0.46, 0.70]	2.11	.019

*Note.* Abd.: Abdomen; Thor: thorax; LR: laughter response; HA: ha responses; Amplitude: trunk compression (circumference reduction) during laughter as a percentage of the resting trunk circumference.

**Figure 4:**
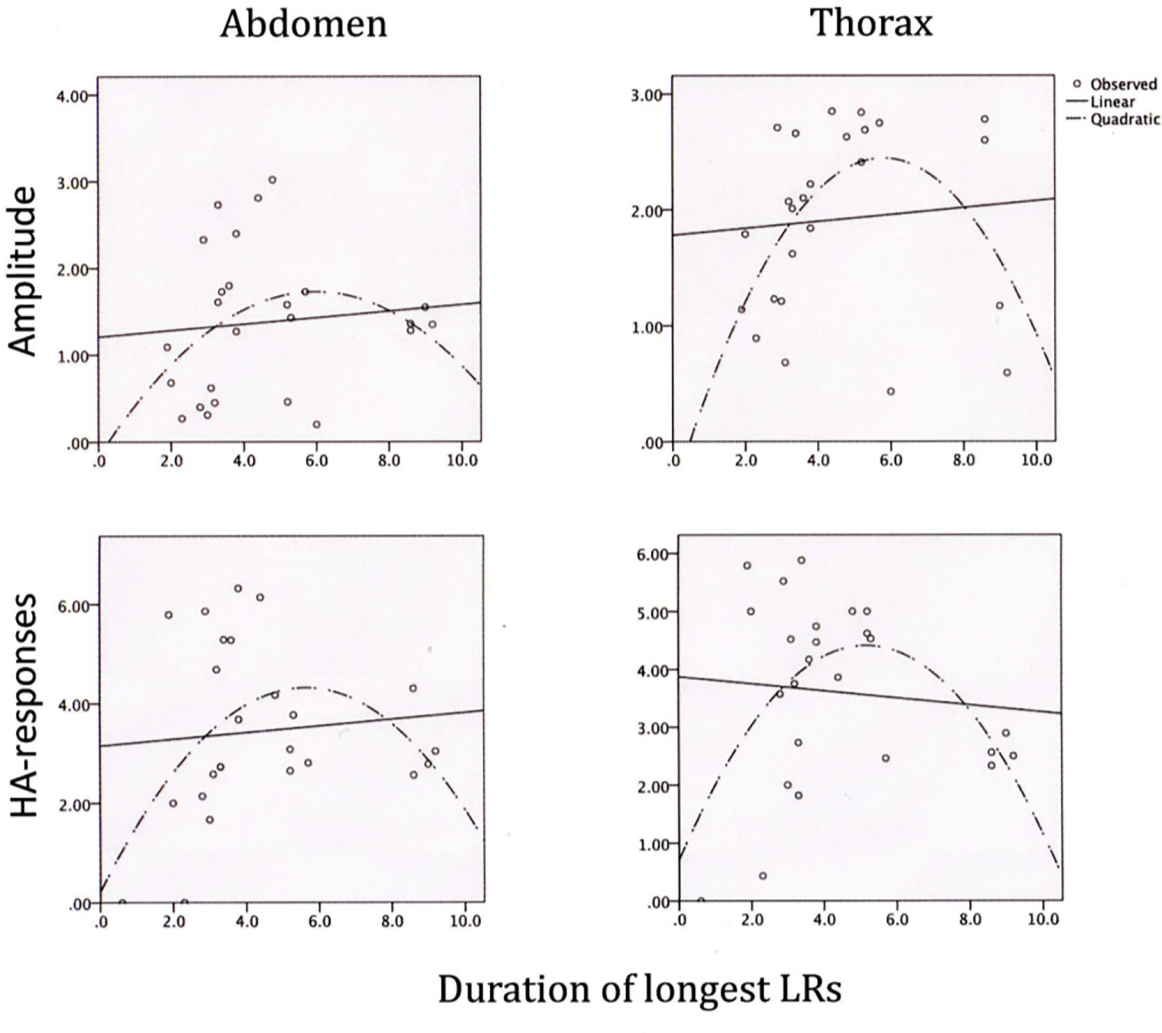
Linear and polynomial relations between (a) the mean duration of the five longest laughter responses and (b) abdominal and thoracic compression amplitudes during laughter (upper panels) as well as frequencies of abdominal and thoracic HA-responses (lower panels).

None of the beta scores for associations of the mean durations of the five longest LRs with the trunk compression parameters were significant in the linear analyses, and 95% confidence intervals enveloped the zero in all cases. However, all were significant in the polynomial analyses (lower section of Table 3). Thus, the amplitude of the abdomen, on average, tended to be high (just below 2% of the abdominal resting circumference), whereas the corresponding compression amplitude of the thorax was around 2.5%, and the greatest amplitudes occurred with moderate durations of LRs (around 5 sec.: see upper section of Figure 4). Correspondingly, the HA-frequencies were at their highest (just above 4/sec.), in the abdomen as well as the thorax, when the duration of the longest LRs were around 5 seconds (lower section of Figure 4).

### Discussion

The total number of LRs was linearly associated with the duration of the longest LRs. On average, the participants laughed four times per minute, and the mean duration of the five longest LRs was between four and five seconds. Consistent support was given in Study 1 for polynomial relations between the number of LRs with all trunk parameters, and this trend was particularly strong for the *duration* of LRs, whereas linear trends with trunk parameters were mostly not supported. The results supported a greater role for the thorax, relative to the abdomen, in the polynomial relations and appeared to be particularly involved in the production of compression amplitudes during laughter. The findings can be taken as supportive to the assumption that in frequent and long LRs, the central nervous system activates an inhibitory influence on the respiratory muscles to induce a protecting buffer against excessive compression in the thorax cavity. The neural substrate for this influence most likely is one or more of the pathways from the prefrontal cortex to the basal ganglia, the thalamus, the hypothalamus and the amygdala as illustrated in Figure 1 of the report by Wild, Rodden, Grodd, and Ruch (2003).

The cognitive activation of the proposed inhibitory mechanism most likely is based upon the evaluation of the amount of humorous potential that is experienced whenever a cognitive humor synergy is perceived (see Apter & Desselles, 2012, for definition of two forms of humor synergies). The mechanism must involve a calculation of the compression potential and the duration of the LR that is expected to follow. In this way the mechanism must act, not as a feedback mechanism, but as a feed forward mechanism. And the protective inhibitory drive appears to be triggered with expectations of LRs beyond around five seconds. Moreover, this inhibitory mechanism may be triggered to suppress any LR for other reasons than to protect against harm to vital organs in the trunk cavity, such as for ethical reasons and for social or personal reasons.

The seemingly secondary role of the abdomen, compared with that of the thorax, in the polynomial dynamics during laughter may be taken as support to a buffering mechanism that primarily aims at protecting the organs of the thorax, the heart and lungs in particular.

Gender differences were due to significantly greater compressions amplitudes in the abdomen of the males than of the females during laughter, and they presented significantly higher HA-frequencies than the females in both the abdomen and the thorax. Moreover, the compression amplitudes for the thorax were significantly greater than for the abdomen in the males as well as the females.

The most parsimonious explanation for the gender differences in trunk mobility during laughter perhaps is the anatomical fact that physical load due to the female breasts acts as a weight constraint to high compression amplitudes and HA-frequencies. This assumption was most consistently supported by the significant difference in the saccadic HA-frequencies where this load may be acting as a greater constraint than in the compression amplitudes of the LRs.

A linear trend was supported in the relation between frequency and duration of LRs. This meant that LRs tended to be longer when they were more frequent (Figure 2). This aspect of overt expressive behavior, however, did not reflect the measures of covert behavior scored as the trunk compression variables. There, in contrast, polynomial relations were particularly strong with the duration scores for the LRs. In this way, the present findings supported a need for the distinction between the overt social behavior and the biological significance of laughter.

Provine (2000: e. g. page 36) argued that several demands act simultaneously upon the breathing apparatus, from other sources than laughter, such as speaking and the continuous need for oxygen. The present polynomial results may be taken also as support to this view. It is possible that the inverted U-shaped relation is present in laughter whether it is induced by humorous amusement or by tickling, and that other concerns than the protection against excessive pressure to the trunk cavity may be activated in all normal causes of laughter. Disregarding the number of potential causes of laughter and the concerns involved, protection against harm and possible adaptive behavioral as well as respiratory and other concerns can be met by an inhibitory brain control mechanism of the muscles involved in vigorous laughter, and the present results emerged as preliminary support to such a mechanism.

## Study 2

The focus in Study 2 is on the consequences of laughter upon the parietal cortex immediately *after* a period of amusement, rather than on brain mechanisms that may protect against harm to vital organs of the trunk cavity *during* laughter. Cortical activation should be expected as a consequence of humor appreciation and be a biological substrate to the subjective experience of refreshment that is often experienced after laughter. The bioelectric and neurochemical nature of this activation in itself involves no risk of biological harm. A linear trend, rather than polynomial, was expected between the number of LRs during exposure to a comedy and the subsequent cortical activation. Alpha power is a classic indicator of a relaxed, but wakeful state, whereas beta power indicates active and conscious processing. Increase of beta power, and decrease of alpha power, were therefore predicted as linear cortical consequences of laughter. These predicted changes would be consistent with the refreshing mental effect often experienced after mirthful laughter.

The limited research in the behavioral and biological parameters of laughter was pointed out by Stillman, Baumeister, and DeWall (2007) who themselves reported on the effects of asymmetrical social power relations in laughter. Conversely, research on the expression of laughter in rats, dogs and primates (Panksepp & Burgdorf, 2000; Ross, Owren, & Zimmermann, 2009), and the related biology, so far has not shed much light upon the brain *consequences* of laughter. Neither has research into the neural brain substrates of laughter expression. Results underline the general importance in humor appreciation of the frontal lobes, the right one in particular (Shammi & Stuss, 1999).

A distinction should be made, for several reasons, between humor appreciation and laughter because laughter is released by tickling where humor appreciation often is not implicated. Also, a number of brain abnormalities can cause the motor behavior of laughter without being accompanied by humorous amusement. Source of laughter notwithstanding, it is dependent upon a complex interplay of many brain areas including the anterior cingulate gyrus, the thalamus, the hypothalamus, the amygdala and other limbic structures that are also involved in a range of other affective displays. However, progress is being made to identify brain areas involved in mirthful laughter, including the supplementary motor areas (Osaka et al., 2003) and the nucleus accumbens, the latter probably being of importance in friendly humor taken its role in empathy (Meeks & Jeste, 2009; Preston & de Waal, 2002).

Recently, cerebellar involvement in cognition and the regulation of the expression of emotions has come into focus, including the control of laughter in depressed patients. However, the prevalent depression, rather than the cerebellar damage, may be of primary importance in the appreciation and expression of humor in these patients (Frank et al., 2013). Obviously, the social effects of contagious laughter, as well as the motor organization of the laughter response, have come more into focus in recent research. The extensive review of neural correlates of laughter as well as humor appreciation by Wild et al. (2003) defined the most significant brain areas in the laughter network to include the prefrontal (medial, dorsolateral) areas, the premotor cortex, basal temporal lobes including the amygdala, the basal ganglia, the thalamus and hypothalamus, the periaquaductus gray, the cerebellum and cranial motor neurons. Brain substrates to laughter were not the target in Study 2. Instead the idea was tested of a cortical substrate of activation after laughter and that, therefore, may explain the vitalizing effect typically experienced after mirthful laughter.

### Method

#### Subjects

Fifty-six undergraduate university students volunteered to take part in the study (31 males; age range: 18-29; mean age: 20.3). They were not compensated for their participation.

#### Apparatus

Respiratory movements were estimated by use of the same technology as described above in Study 1. Monopolar left (P3) and right (P4) parietal EEG-recordings were derived by means of Beckman EEG-electrodes located on the parietal surface according to criteria given in the 10-20-system (Cooper, Osselton, & Shaw, 1980). A joint reference lead was applied to the right and left ear lobes. Resistance between this joint reference lead and each of the active sites was below 2 Kohm. Bentonite paste was used on both active sites, and the Beckman electrode paste was used at the ear lobes. The power spectrum scores were computed on-line for each of the alpha and beta bands by means of a Hewlett Packard 2100A computer with a sampling rate of 128/second.

#### Materials, Procedure and Statistical Analyses

The subject was seated in an electrically shielded and sound-proof compartment (2.5 by 3.5 m Tegnér, Stockholm). A two-way communication system was used for instructions. Right and left power spectrum scores were computed on-line for each of the alpha (8-12 Hz) and beta (13-30 Hz) bands and for each hemisphere.

The subjects were exposed one by one to the classic comedy “Dinner for one” that lasted for 12 minutes. It was presented on a Philips TC 1655 UR Television screen by means of a Panasonic NV 870 video recorder with sound transmission via a NAD 3020 B power unit and Beovox C 30 loudspeakers. The television screen covered the window of the compartment and was located at the outside.

The subject was instructed to relax with closed eyes for 10 minutes before the video program, and for 2-minutes immediately after the termination of the program. Resting with closed eyes in both baselines meant that EEG artifacts due to eye movements were assumed to be minimal and symmetrical across the pre- and post-baseline recordings. Pre- and post-baseline power spectrum scores were computed over eight consecutive 8-second periods in the baseline periods, immediately before start of the comedy and at its termination. Scores were summed across the hemispheres and for each frequency band. This meant that four power scores were computed for each subject. In the statistical analyses, difference scores for the pre-to-post comedy exposure change of power were computed for the alpha and beta bands. Regression analyses were performed by use of the JMP software to test associations between number of laughter responses over the 12 minutes of comedy exposure and changes in power for the alpha and beta bands, respectively.

### Results

**Figure 5:**
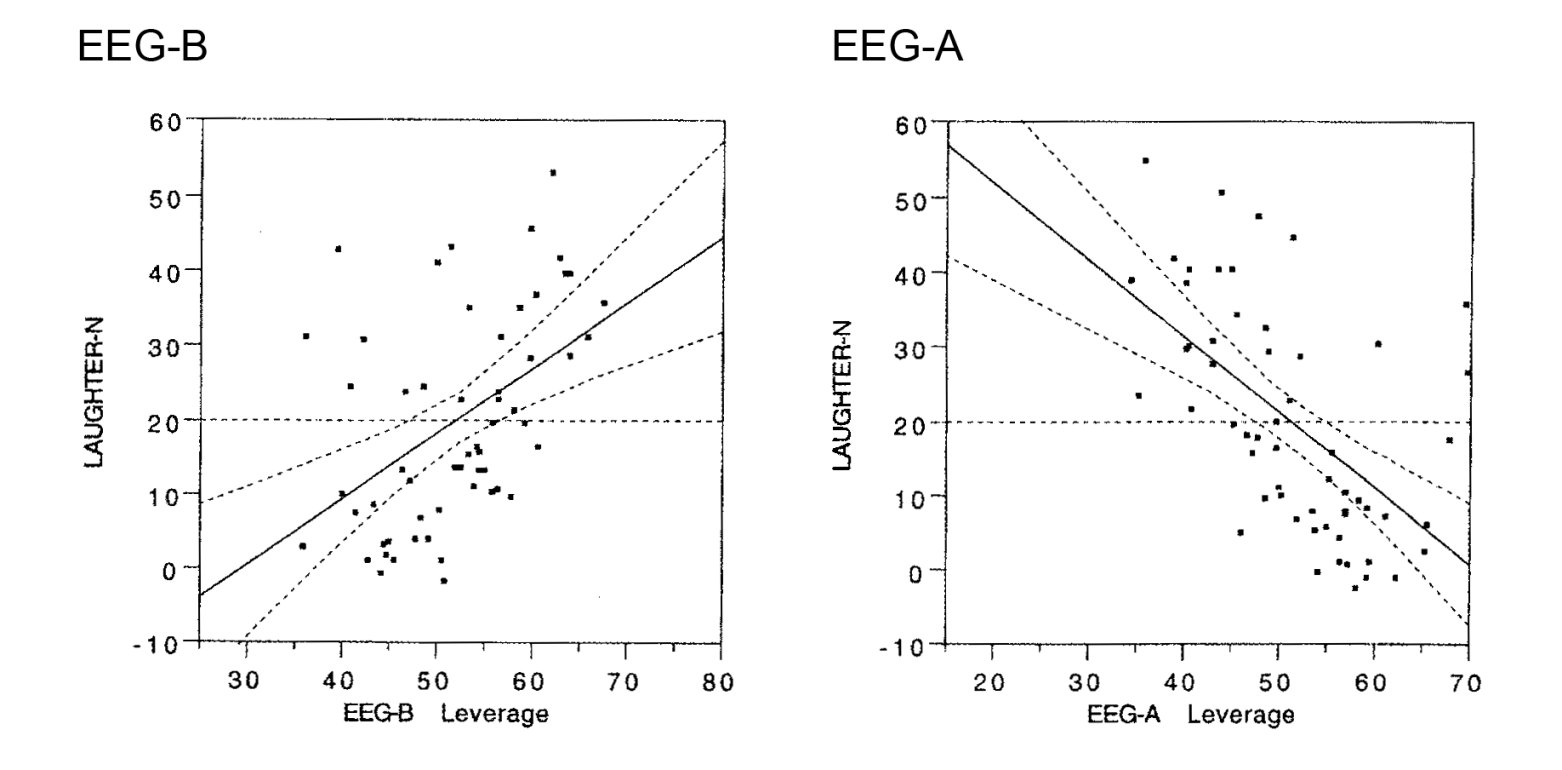
Changes in parietal cortex EEG power (pre-to-post comedy change scores) as consequences of laughter during exposure to a comedy (“Dinner for one”). Left panel: Beta band (EEG-B). Right panel: Alpha band (EEG-A). The 95% confidence intervals are given as dotted lines around the regression lines.

Laughter responses ranged from 0 to 54 over the 12-minute comedy exposure. No gender difference was seen, and both parietal lobes contributed jointly to the power scores in the pre- as well as post-sampling period. It will be seen in Figure 5 (left panel) that power scores for the beta band increased substantially from pre- to post comedy exposure, and this increase was strongly associated with the number of laughter responses. A marked linear trend can be seen due to high power scores after the comedy among those who responded with frequent laughter. The F-score for this linear test was 15.92 (df = 1/54; p < .0002; 95% CI [0,14, 0,43]; model fit: beta = 0.29). An even stronger, but opposite, trend was calculated for the alpha band due to strong reductions of alpha power after the comedy exposure among those with frequent laughter. The F-score for this linear change was 26.71 (df = 1/54; p < .00004; 95% CI [0,47, 0,21]; model fit: beta = 0.34). This trend is illustrated in the right panel of Figure 5. The 95% confidence interval curves are illustrated with dotted lines in this figure. They are almost parallel to the regression lines and thus also support highly stable models (see Privitera, 2016, p. 438).

### Discussion

The results in Study 2 supported the predicted linear trends for cortical activation after laughter. The beta power was strongly increased as a function of number of laughter responses. Correspondingly, the alpha power of the parietal lobes was reduced as a linear function of frequent laughter after exposure to the comedy. In this way, the results of Study 2 supported the conclusion that mirthful laughter activates the cortex of the brain and, therefore, may facilitate subsequent cognitive processing.

The results should not be taken as conclusive evidence in support of a general effect across the hemispheres and lobes in light of the fact that recordings were derived from the parietal lobes exclusively. Moreover, artifacts due to eye movements cannot be ruled out despite the fact that power measures were derived with eyes closed an under identical resting conditions before as well as after exposure to the comedy. However, the highly significant model fits for the beta as well as the alpha power scores with number of LRs provide strong preliminary support to the conclusion of cortical activation after laughter. Eye movements most likely appeared as random artifacts in the resting periods before as well as after exposure to the comedy.

## Summary and Concluding Discussion

The results in Study 1 supported the idea of an inhibitory brain mechanism that may be triggered to protect the trunk in general, and the thorax cavity in particular, from excessive compression due to enduring laughter (beyond 5 sec.). In Study 2, results supported a cortical substrate for the experience of mental refreshment after laughter. There was a shift in cortical activation after comedy exposure, and this was seen both in the beta and alpha power changes from the pre- to the post-comedy resting period.

Both findings are preliminary in nature and the results from Study 1 were obtained by use of a correlational design, whereas the results in Study 2 were based upon the use of a combined correlational and pre-to-post predictive design. In Study 1, gender of the participant experimenter was not balanced across gender of the subjects and, therefore, may have influenced the gender differences. In both experiments, two Hg-gauges were applied, one at the level of the armpits and the other at the level of the navel. This means that not the whole trunk was monitored during compression due to laughter.

The expiratory nature of trunk compression, when repeated in frequent laughter, such as in the two present studies, may cause a relative shift toward alkalosis that has been known for several decades to cause a diffuse excitation in all nervous tissue, including the central nervous system. Frequent laughter is a potential cause of (mild) alkalosis induced by laughter and, therefore, can explain the parietal excitatory shift from pre- to post-exposure in Study 2. Acute alkalosis can be triggered by several causes including a panic attack and acute performance stress anxiety. Alkalosis has mostly been investigated in relation to hyperventilation and panic disorders. Research on causes of moderate shifts toward alkalosis may call for a wider causal context including a possible shift from acidosis to alkalosis following laughter. Unfortunately, there appears to be no evidence published to shed light upon this possibility. Most of this research still has a focus upon clinical problems such as panic and performance stress anxiety (e.g. Meuret, Wilhelm, Ritz, & Roth, 2008; Studer et al., 2012).

In his milestone analysis of the expression of emotions in man and animals, Darwin (1872/1965) presented an extensive analysis of the expression of laughter with a focus on the muscles of the face. However, on page 200 he describes the role of the respiratory apparatus where “…laughter is produced by a deep inspiration followed by short, interrupted, spasmodic contractions of the chest, and especially of the diaphragm.” The present findings, and those reported by Filippelli et al. (2001), did not support the initial inspiratory phase, proposed by Charles Darwin, because most of the trunk circumference changes in laughter started with a marked expiratory movement that exerts the acute increase of pressure in the trunk cavity (see Figure 1). A primary role of the diaphragm can also be disputed due to relatively greater compression amplitudes of the chest in laughter, when compared with those of the abdomen (on average 1.4 versus 1.9% circumference reduction, respectively) according to the results in Study 1. In contrast, the intercostal muscles of the rib cage appeared to be particularly active during laughter. With due respect, sensitive technology was not available for measurement of trunk involvement in spontaneous laughter around 1870.

Recently, EMG measures were reported of front and back muscle tension during laughter yoga (Wagner, Rehmes, Kohle, & Puta, 2014). The involvement of five muscles (front: internal and external obliques, rectus abdominis; back: multifidus, erector spinae) was compared with their tension during the performance of traditional crunch and back lifting exercises. Tension was high in the internal oblique muscles during laughter yoga, whereas the external oblique muscle tension was identical to that of the traditional exercises. The EMG surface electrodes were not sensitive to tension changes in the diaphragm and the intercostal muscles of the rib cage. The internal intercostal muscles are of particular importance in forced expiration, and the present results extended their role to also include vigorous laughter. Also, the fact that laughter was induced by yoga instructions, and not by amusement in humor, may have left these findings with poor validity for motor involvement in spontaneous laughter. Furthermore, intense laughter may sometimes induce spasms in the muscles of the extremities that interfere with normal standing or sitting positions. Other intense emotional expressions may also induce altered body positions. Regardless of the source, the related cascade of muscle tension is not readily conveyed in participant observation.

The present measurement technology has proven to be highly sensitive to trunk circumference changes and can easily detect changes below one millimeter both in the thorax and the abdomen (Svebak, 1986). Despite this advantage, it is a time consuming technology to apply, and it involves the need for production of many gauges with different lengths to allow for between 25 and 35 percent of stretching to assure accurate measurements when in use in participants with a range of trunk sizes. Also, the elaborate calibration procedure at the end of a laboratory session, for every participant, provides the absolute metric scale for amplitude changes relative to the resting circumference at both trunk levels. However, the most obvious advantage of this Hg-gauge technology probably is the non-invasive nature and the lack of sensory distraction to the subject when in use.
